# Physicochemical Properties and Microbial Quality of *Tremella aurantialba* Packed in Antimicrobial Composite Films

**DOI:** 10.3390/molecules22030500

**Published:** 2017-03-22

**Authors:** Jian Fan, Zhuangzhuang Chu, Lin Li, Tianrui Zhao, Min Yin, Yuyue Qin

**Affiliations:** 1 Institute of Yunnan Food Safety, Kunming University of Science and Technology, Kunming 650550, Yunnan, China; fanj333@163.com (J.F.); 18487262163@163.com (Z.C.); food363@163.com (T.Z.); m18468259476@163.com (M.Y.); 2 College of Light Industry and Food Science, South China University of Technology, Guangzhou 510640, Guangdong, China; felinli@scut.edu.cn

**Keywords:** poly(lactic acid), nano-TiO_2_, nano-Ag, *Tremella aurantialba*, physicochemical quality, microbial quality

## Abstract

The effects of poly(lactic acid) (PLA)-based film with inorganic antimicrobial nano-TiO_2_ and nano-Ag on the physicochemical and microbial quality of *Tremella aurantialba* stored at 4 ± 1 °C for 16 days was investigated. Rosemary essential oil (REO, 9 wt %) was added into PLA film as plasticizer. Low-density polyethylene (LDPE) and PLA film was used as the controls. The experiment measured physicochemical properties and microbial levels, such as weight loss, firmness, vitamin C, color, microbiological quality, and sensory quality. Although *Tremella aurantialba* packed by nano-composite films had the highest weight loss (4.96% and 5.17%) at the end of storage, it was still in the vicinity of 5%. *Tremella aurantialba* packed with nano-composite films were significantly (*p* < 0.05) firmer than those packed by LDPE, PLA, and PLA/REO films. The nano-composite films were more effective in reducing vitamin C and microbial counts and preserving the color of *Tremella aurantialba* than the other three groups. The overall acceptability of *Tremella aurantialba* packed by the nano-composite films still remained good and within the limits of marketability after 12 days of storage. The results suggested that the proposed nano-composite films could maintain the quality of *Tremella aurantialba* and extend its postharvest life.

## 1. Introduction

*Tremella aurantialba*, belonging to the Basidiomycetes and the order Tremellales, is an edible and medicinal jelly fungus with a brain-shaped, golden and large basidiocarp [1]. *T. aurantialba* is exquisite and delicious, having the medicinal effects of resolving phlegm, relieving coughs, stopping asthma and regulating Qi, and a high nutritional value. *T. aurantialba* is mainly distributed at an altitude of 1000–3000 m in regions like Tibet, Yunnan, Sichuan, and Gansu Province, where it grows on the decaying wood of broad-leaved trees like *Quercus aquifolioides* in the summer and autumn. *T. aurantialba* is unfortunately highly perishable. Due to the absence of a cuticle to protect the mushroom from microbial attack or water loss, numerous changes in the counts of bacteria, mould, enzymatic activities, and biochemical changes would cause spoilage [2,3,4,5]. The short shelf-life of *T. aurantialba*, typically one to three days at ambient temperature, is an impediment to the distribution and marketing of the fresh produce. Nevertheless, there has almost no research on the postharvest handling of *T. aurantialba*.

Poly(lactic acid) (PLA) is biocompatible and undergoes scission in the body to lactic acid, which can be produced from the fermentation of annually renewable resources, like sugar beet or corn starch [6,7]. PLA has been approved by the United States Food and Drug Administration (FDA). Due to its relatively low cost, processibility as well as biocompatibility, PLA is an interesting candidate for producing food packaging materials [7,8,9]. However, the large amount of crystallinity in PLA results in it having not only a high modulus and strength, but also a brittle nature. In our previous study, we have tried to prepare PLA composite film with 9 wt % rosemary essential oil (REO) as plasticizer. PLA/REO composite film can improve upon the flexibility and toughness properties of PLA [10]. The natural plant essential oil are categorized as GRAS by the FDA as well as the current European legislation for materials intended to be in contact with foodstuffs [11].

In recent years, some new types of nano-inorganic antimicrobial materials, such as nano-TiO_2_ and nano-Ag, have been widely used in many fields for their antimicrobial properties. Nano-TiO_2_ has the advantages of high photoreactivity, cheapness, non-toxicity, and chemical stability, so it is promising for eliminating microorganisms in self-cleaning and self-sterilizing materials [12]. Nano-Ag is a promising antimicrobial material. Silver nanoparticles can attach to the cell membranes and penetrate into bacteria. They have been widely used in food packaging, water filtration, textiles, and health care due to their outstanding antimicrobial properties [13]. Nano-TiO_2_ and nano-Ag have both been approved by the FDA for use in the food industry [13,14].

In this work, *T. aurantialba* was packed in a PLA nano-composite film, and the effect of the films on the physicochemical and microbial quality of *T. aurantialba* during refrigerated storage was investigated. Several variables of *T. aurantialba* were evaluated during 16 days of storage, among which were the weight loss, tissue firmness, vitamin C, color, microbiological quality, and sensory attributes.

## 2. Results and Discussion

### 2.1. Weight Loss

**Figure 1:**
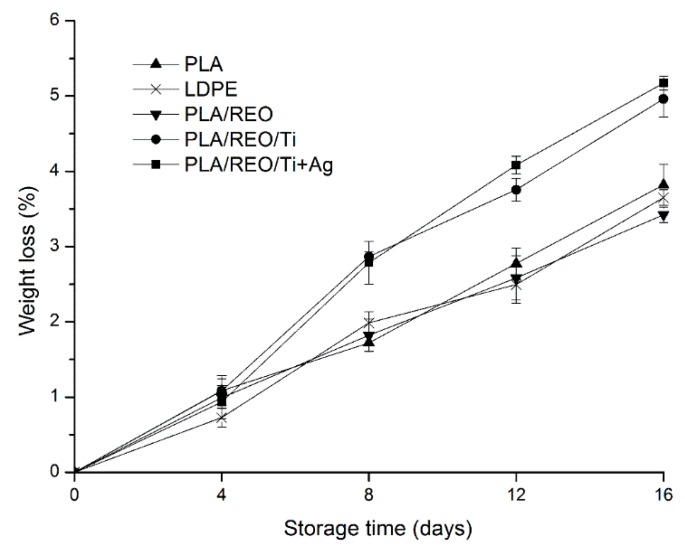
Effect of different packages on the weight loss of *T. aurantialba* stored at 4 ± 1 °C for 16 days. Data are presented as mean ± standard deviation.

The weight loss from packed edible fungus, that includes dry matter and moisture, depends on the postharvest respiration and transpiration in fresh samples, and the microbial growth and water vapour barrier properties of any packaging [5]. The weight loss of *T. aurantialba* during storage is shown in Figure 1. As can be seen, the weight loss increased with the duration of storage for all samples. At the end of the storage, the weight loss was 3.82%, 3.65%, 3.42%, 4.96%, and 5.17% for PLA, LDPE, PLA/REO, PLA/REO/Ti and PLA/REO/Ti + Ag, respectively. This might be because that harvested *T. aurantialba* were only protected by a thin film which could not prevent the loss of moisture caused by a high transpiration rate, and dry matter and gas through respiration. The weight loss of *T. aurantialba* packed in PLA/REO/nano composite films was significantly (*p* < 0.05) higher than that samples packed in other three films after 8 days of storage. The maximum weight loss was recorded for samples packed in the PLA/REO/Ti + Ag film at the end of storage, which had higher water vapor permeability than LDPE, PLA, PLA/REO, and PLA/REO/Ti films. The water vapor permeability of the PLA, LDPE, PLA/REO, PLA/REO/Ti, and PLA/REO/Ti + Ag film was 1.833 × 10^−13^, 1.433 × 10^−13^, 1.128 × 10^−13^, 3.932 × 10^−13^, and 5.656 × 10^−13^ kg∙m/m^2^∙s∙Pa, respectively. The increase in water vapor permeability of PLA/REO/nano composite films could be because many micropores exist in the composite films and permit more water vapor transfer [15]. There was no significant (*p* > 0.05) difference between the weight loss of samples packed in PLA, LDPE, PLA/REO film, which was indiated by a similar water vapor permeability.

When a weight loss of 5%–10% of postharvest mushroom happens, the mushrooms begin to wilt and soon become unusable. In this experiment, the highest weight loss (5.17%) was found in the PLA/REO/Ti + Ag treatment at the end of storage, which represented a slight effect on the commercial value of *T. aurantialba* [16].

### 2.2. Firmness

**Figure 2:**
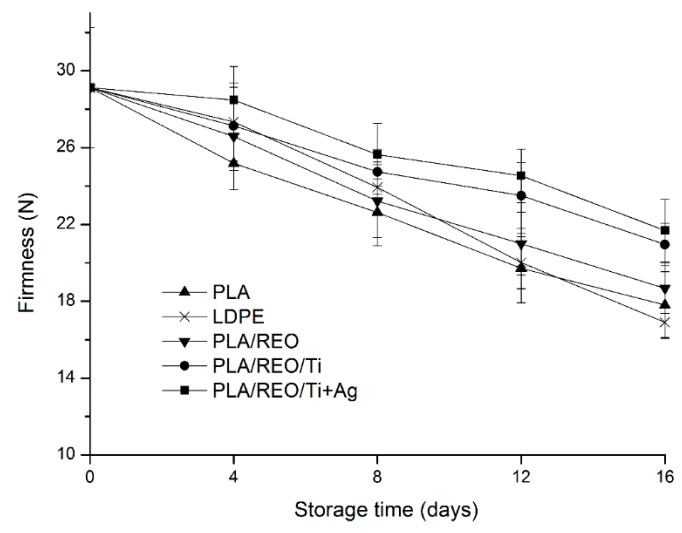
Effect of different packages on the firmness of *T. aurantialba* stored at 4 ± 1 °C for 16 days. Data are presented as mean ± standard deviation.

Texture is one of the important quality indexes of fresh edible fungi. Excessive softening may lead to shrinkage, drying, and biochemical changes, and even reduce the quality and acceptability of the fresh produce [17]. Postharvest fresh *T. aurantialba* was of a hardish, brittle, and tender quality, but after a period of unpackaged storage, the quality of *T. aurantialba* obviously declined. The firmness of *T. aurantialba* during storage is shown in Figure 2. The firmness of samples packed with all investigated films decreased during the storage period. This might be because of the degradation of proteins and polysaccharides, shrinkage of the mycelium, and rupture of the central vacuole [18]. After 16 days of storage, the firmness of samples declined from 29.13 N to a range of 16.91 N–21.68 N.

The samples packaged in the PLA/REO/nano composite films displayed significantly (*p* < 0.05) higher firmness than those packed in PLA, LDPE and PLA/REO films. This could be due to a slower rate of dehydration, conserving the firmness of the surface of samples [19]. On the other hand, the water vapor permeability of nanocomposite films was higher than that of the other three films. The packaging films with low permeability rate could promote the relative humidity within the package, which in turn speeds up the process of edible fungus softening [18]. Therefore, *T. aurantialba* packed in PLA/REO/Ti + Ag and PLA/REO/Ti composite films with a higher water transmission rate were firmer than with the other three films.

### 2.3. Vitamin C

**Figure 3:**
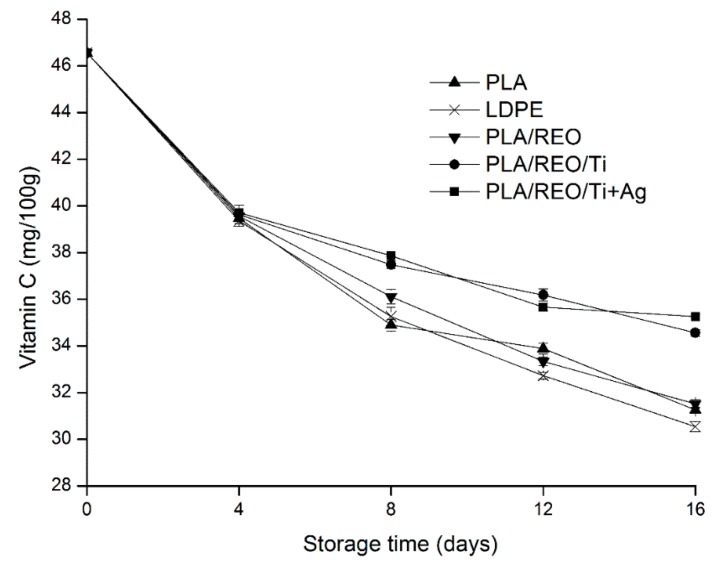
Effect of different packages on the vitamin C level of dry *T. aurantialba* stored at 4 ± 1 °C for 16 days. Data are presented as mean ± standard deviation.

Vitamin C is an important nutrient in fruits and vegetables, which is used to measure an important index of edible value of fungi. The vitamin C content in edible fungi is not high, and vitamin C is very vulnerable to oxidation by enzymes and metal ions. In addition, improper storage processes will also accelerate the vitamin C content reduction [20]. The vitamin C content of *T. aurantialba* during storage is shown in Figure 3, where the vitamin C content decreased with the duration of storage for all dry samples. This might be because of the consumption by the physiological metabolism and oxidation of *T. aurantialba*. Vitamin C content declined linearly and there was no significant difference (*p* > 0.05) in the content of dry samples packed by all films during the first 4 days of storage. However, the content of vitamin C had a smooth downward trending curve. The content of dry samples packed by films with nanoparticles was higher than that packed in the other films. This could be due to the addition of nano-TiO_2_ and nano-Ag, which would change the membrane permeability, inhibit the respiration, and reduce the consumption of vitamin C content to some extent [17]. At the end of storage, the vitamin C content of dry *T. aurantialba* packed in PLA/REO/Ti and PLA/REO/Ti + Ag films was 34.57 and 35.27 mg/100 g, so compared with the initial content it had decreased by 24.26% and 25.76%, respectively, so the ability of PLA/REO/nanocomposite films to maintain the vitamin C content in *T. aurantialba* was superior to that of PLA, LDPE, PLA/REO film packages.

### 2.4. Color

**Figure 4:**
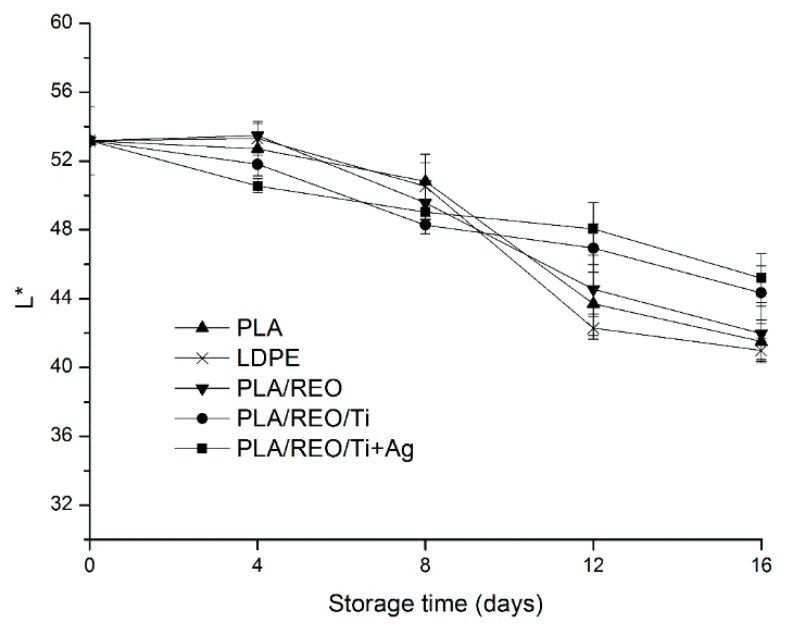
Effect of different packages on the L* value of *T. aurantialba* stored at 4 ± 1 °C for 16 days. Data are presented as mean ± standard deviation.

**Figure 5:**
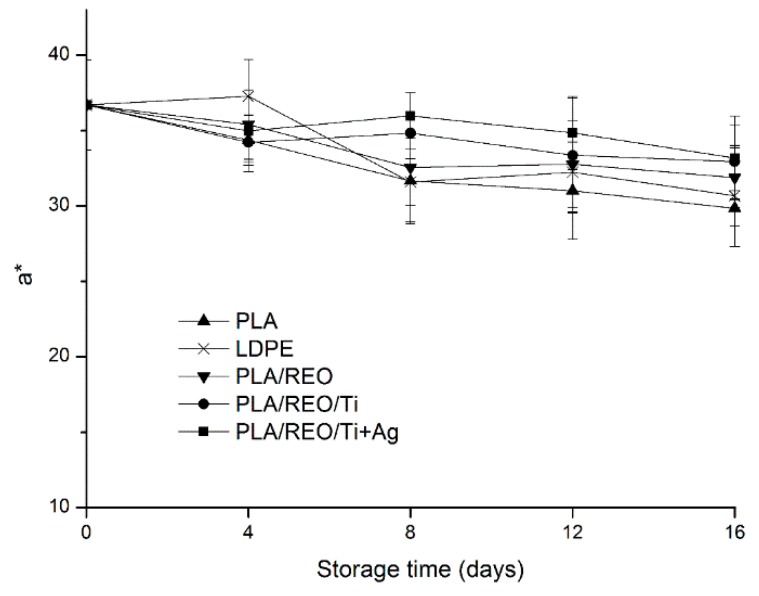
Effect of different packages on the a* value of *T. aurantialba* stored at 4 ± 1 °C for 16 days. Data are presented as mean ± standard deviation.

**Figure 6:**
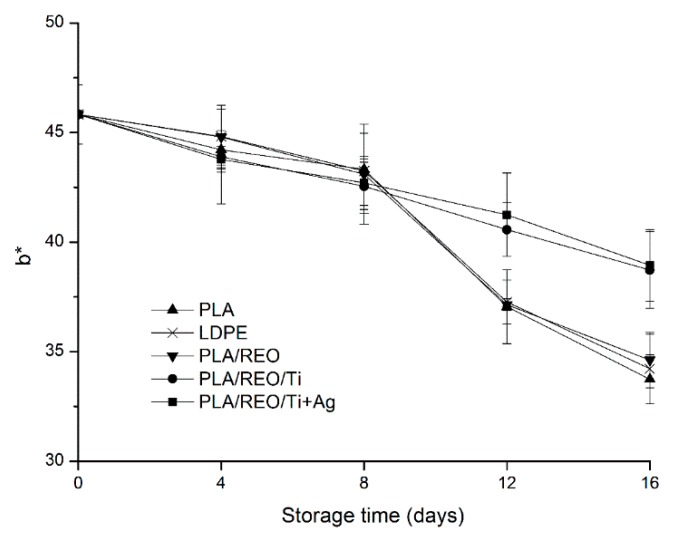
Effect of different packages on the b* value of *T. aurantialba* stored at 4 ± 1 °C for 16 days. Data are presented as mean ± standard deviation.

To consumers, the appearance of the food is one of the main criteria to buy or not [21], and color is the first food quality attribute evaluated by consumers. The changes of color in L*, a*, and b* values during storage are shown in Figure 4, Figure 5 and Figure 6.

L* value depends on the reflectivity, which is used to express the luminosity of the surface of samples. The lower the L* value, the darker the surface of samples [22]. As shown in Figure 4, the L* value of samples decreased from 53.17 to a range of 40.99–45.19 at 16 days of storage, which indicated that the color of the surface in *T. aurantialba* packed in the five films was darker. The L* value of samples packed in PLA/REO/Ti and PLA/REO/Ti + Ag composite film were significantly (*p* < 0.05) higher than with the other three treatments after 12 days of storage. The a* values of all samples decreased during the whole storage period. This suggested that the color of samples transformed the red into the light green. As expected, the a* value of samples packed in PLA/REO/Ti and PLA/REO/Ti + Ag composite film were significantly (*p* < 0.05) higher than with the other three treatments. The b* value is a critical parameter of *T. aurantialba* during the storage period, because *T. aurantialba* contains lots of yellow pigments. As can be seen in Figure 6, the b* value of all samples decreased during the storage time. There was no significant difference (*p* > 0.05) in the b* values of samples packaged in between PLA/REO/Ti and PLA/REO/Ti + Ag films at 12 days of storage.

The b* values of samples packed by the nanocomposite films were significantly (*p* < 0.05) higher than with the other three treatments from day 12 to the end of storage. This might be because nanocomposite film inhibits the reduction of the enzyme which could synthesize the yellow pigments [23].

### 2.5. Microbiological Analysis

**Figure 7:**
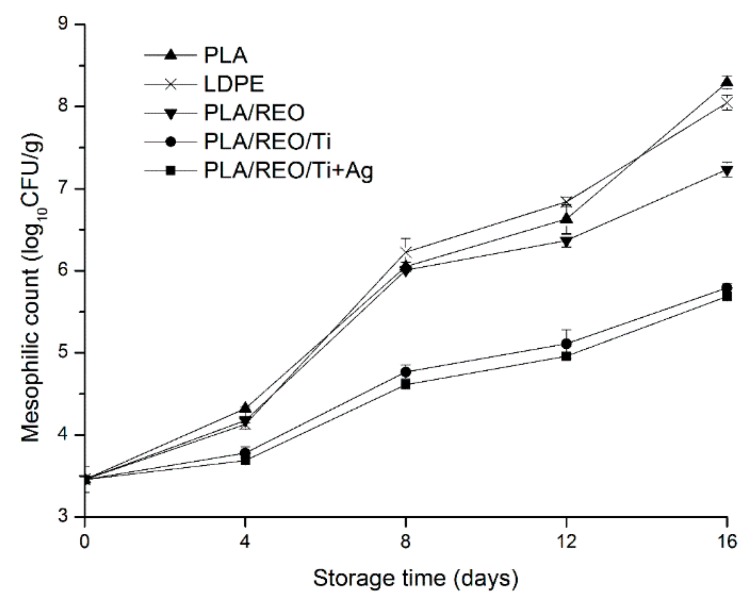
Effect of different packages on the mesophilic counts of *T. aurantialba* stored at 4 ± 1 °C for 16 days. Data are presented as mean ± standard deviation.

**Figure 8:**
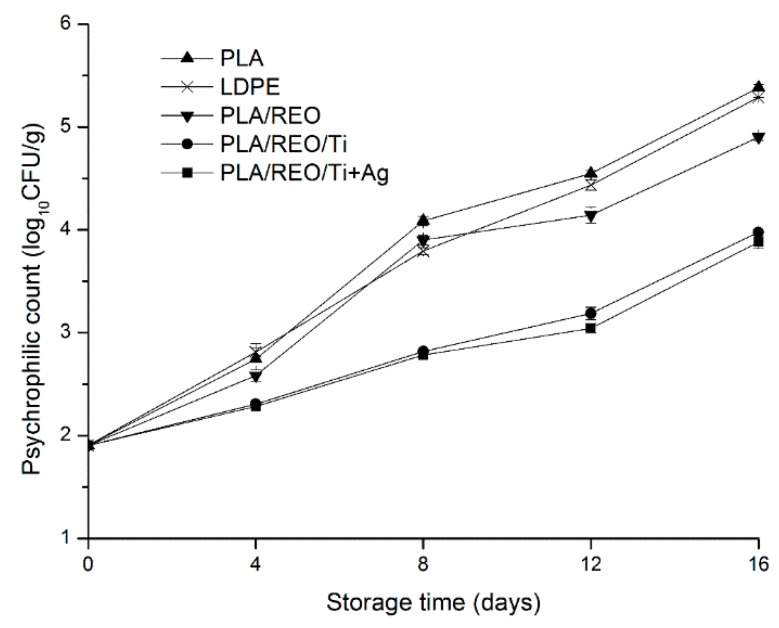
Effect of different packages on the psychrophilic counts of *T. aurantialba* stored at 4 ± 1 °C for 16 days. Data are presented as mean ± standard deviation.

Edible fungi are vulnerable to few bacterial (such as mesophilic and psychrophilic bacterial) diseases, probably due to defense mechanisms that have evolved through living in a rotting environment [24]. The changes of total aerobic bacteria count of *T. aurantialba* during storage are shown in Figure 7 and Figure 8. In all of the treatments, the total mesophilic and psychrophilic bacteria gradually increased during the whole storage period. At the same storage time, the mesophilic bacteria count was higher than the psychrophilic bacteria count. This could be because that most microbes grow and reproduce under moderate conditions.

The PLA/REO film showed significantly (*p* < 0.05) higher antimicrobial activity than the PLA and LDPE films at the end of storage. Antimicrobial components were not incorporated into PLA and LDPE film, so the pure PLA and LDPE films do not inhibit the growth of microorganisms. The antimicrobial properties of REO are mainly related to phenol diterpenes, such as carnosol, carnosic acid, rosmanol, rosmarinic acid, and isorosmanol [25]. The addition of REO decreased the glass transition temperature of PLA/REO blends and REO was used as plasticizer for PLA polymer. PLA/REO film inhibited both *E. coli* and *B. subtilis* due to the inhibition ability of REO [10].

The total mesophilic and psychrophilic bacteria of samples packed by the PLA/REO/Ti and PLA/REO/Ti + Ag composite film showed significantly (*p* < 0.05) lower levels than those in PLA, LDPE, PLA/REO film. There was no significant difference (*p* > 0.05) in the total bacteria between PLA/REO/Ti and PLA/REO/Ti + Ag groups during the whole storage time. As expected, the incorporation of nano-TiO_2_ or nano-Ag into the polymer matrix could effectively inhibit the growth of microorganisms in *T. aurantialba*. The antimicrobial mechanism of nano-Ag is related to membrane damage due to free radicals derived from the nano-Ag surface [26]. Nano-TiO_2_ could kill microbes such as *E. coli* and *Candida albicans* [27]. Due to the small particle size of TiO_2_, more electron-hole pairs were generated which induced redox reactions on those microorganisms [28]. Furthermore, microbial species would easily grow in a humid environment, so the PLA/REO/Ti and PLA/REO/Ti + Ag composite films with their high permeability rates could effectively inhibit the microbial growth.

### 2.6. Overall Acceptability

**Figure 9:**
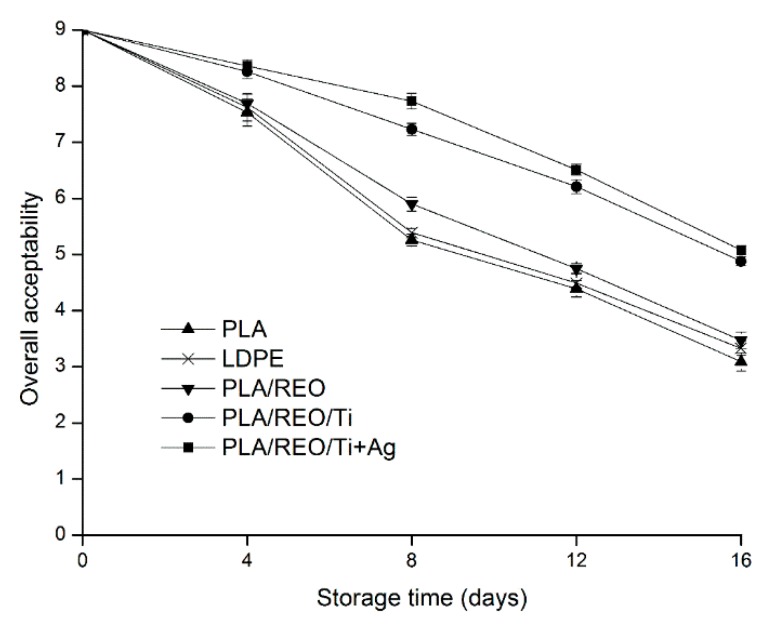
Effect of different packages on the overall acceptability of *T. aurantialba* stored at 4 ± 1 °C for 16 days. Data are presented as mean ± standard deviation.

The overall acceptability of *T. aurantialba* during storage is shown in Figure 9. As shown, the overall acceptability of *T. aurantialba* gradually decreased as the storage period advanced with all the treatments. According to the evaluation from all panelists, samples packed by PLA/REO/Ti + Ag film had the highest score, and the next was the samples packaged in PLA/REO/Ti film during the storage period. The indexes of overall visual quality, aroma and colour of samples packed in PLA, LDPE, PLA/REO film changed and the score of overall acceptability of samples packed by these film was 4.39, 4.49, and 4.75, respectively, which explained *T. aurantialba* treated by three films were unacceptable after 12 days of storage. However, samples packaged in PLA/REO/Ti and PLA/REO/Ti + Ag film were merely acceptable, with overall acceptability scores 4.88 and 5.08, respectively, even on day 16. This results indicated that the PLA/REO/Ti film and PLA/REO/Ti + Ag film treating could be effective in maintaining the overall acceptability of *T. aurantialba*.

## 3. Materials and Methods

### 3.1. Materials

*T. aurantialba* samples were harvested from a local field in Kunming, Yunnan Province, China. The fresh samples were selected based on their uniformity of shape, maturity, without plant diseases and insect pests and free from mechanical damage. Poly(L-lactic acid) (PLA) was purchased from Natureworks LLC (Blair, NE, USA), nano-TiO_2_ from Xieqing Industrial Co., Ltd. (Shanghai, China), nano-Ag from Wanjing New Material Co., Ltd. (Hangzhou, China), rosemary essential oil (REO) from Dream Castle Electronic Commerce Co., Ltd. (Tianjin, China).

PLA, PLA/REO, and PLA/REO/nano-composite films were prepared by the solvent volatilization method. Prior to blending, the PLA was dried in vacuum at 80 °C for 24 h to eliminate the influence of water. PLA (2 g) and REO (9 wt %) were dissolved in dichloromethane (50 mL) and stirred for 12 h. Then, nanoparticles were added to the PLA/REO dichloromethane solution and stirred by a magnetic stirrer until all of the nanoparticles were completely mixed with the solution. Finally, all the solutions were poured onto polytetrafluoroethylene (PTFE) plates and dried under vacuum. By optimizing the conditions of the pre-test, we decided to add 2 wt % nano-TiO_2_ and 1.5 wt % nano-TiO_2_ plus 0.5 wt % nano-Ag to PLA/REO solution. The PLA film with 9 wt % REO was named PLA/REO film. The PLA film with 9 wt % REO and 2 wt % nano-TiO_2_ was named PLA/REO/Ti nanocomposite film. The PLA film with 9 wt % REO, 1.5 wt % nano-TiO_2_ plus 0.5 wt % nano-Ag was prepared as previous described and named PLA/REO/Ti + Ag nanocomposite film. Low density polyethylene (LDPE) film was purchased from Xingnong Co., Ltd. (Zhongshan, China) and used as control. The water vapor permeability of films was determined gravimetrically in accordance with the ASTM E96-95 standard method [10].

### 3.2. Sample Preparation

*T. aurantialba* samples were randomly divided into five different treatments. Samples of approximately 150 g were weighed and packed into rectangular bags (150 mm × 300 mm) that were constructed from the tested films and stored at 4 ± 1 °C for 16 days. Five groups of samples were prepared in total: PLA group, PLA/REO group, PLA/REO/Ti group, PLA/REO/Ti + Ag group, and LDPE group. Samples were evaluated for physicochemical and microbial quality (weight loss, firmness, vitamin C, color, microbiological quality, and sensory attributes) at 4 day intervals (0, 4th, 8th, 12th, and 16th day).

### 3.3. Weight Loss

The weight of *T. aurantialba* from each package was determined on the initial day and each sampling day. Weight loss was determined gravimetrically. Values were expressed as percentage of weight loss per initial sample weight:

(1) $$\text{Weight loss}\;\left(\%\right)=\frac{M_{0}-M_{1}}{M_{0}}\times 100$$

where *M*_0_ was the weight on the first day and *M_1_* was the weight on each sampling day [15].

### 3.4. Firmness Measurement

The firmness of *T. aurantialba* was determined by a penetration test, using a texture analyser (Texture Exponent 32, Stable Micro Systems Ltd., London, UK) equipped with a cylindrical probe of 2 mm diameter. The sample was penetrated 5 mm in depth. The speed of the probe was fixed at 2 mm∙s^−1^. From the force versus time curves, firmness was defined as the maximum force (Newton, N) [15].

### 3.5. Vitamin C

Vitamin C content of *T. aurantialba* was determined by a spectrophotometric method [29]. The absorption of the vitamin C standard substance was determined from 220 nm to 320 nm, and the wavelength where the absorption peaks was recorded as the maximum absorption wavelength. The absorbance of all samples was measured at 267 nm by UV-vis Spectrophotometer (UV-1800, Mapada Instruments Co., Ltd., Shanghai, China), and then the vitamin C content of samples was calculated from a standard curve [17]. Vitamin C content was expressed as mg of vitamin C content per 100 g of dry sample (mg/100 g).

### 3.6. Color

The surface color of *T. aurantialba* was evaluated by measuring L* (light/dark), a* (red/green), and b* (yellow/blue) using a colorimeter (WSC-S; Shanghai Precision Instrument Co., Ltd., Shanghai, China). Each sample was measured at three equidistant points, and five samples were analysed for each group [17].

### 3.7. Microbiological Analysis

All samples were analyzed for their mesophilic and psychrophilic bacteria counts. *T. aurantialba* (25 g) was removed aseptically from the package, and homogenized in a sterile stomacher bag for 2 min with 225 mL of buffered 0.1% peptone water. Then, 10-fold serial dilution was prepared for the following determination. Aerobic counts were determined on plate count agar. The plates were incubated at 37 °C for 2 days for mesophilic bacteria and at 4 °C for 7 days for psychrophilic bacteria [2]. The results were expressed as log_10_ CFU/g of sample.

### 3.8. Overall Acceptability

The overall acceptability of samples was evaluated according to their overall visual quality, aroma, and colour by a sensory panel of ten assessors from the Institute of Yunnan Food Safety, Kunming University of Science and Technology. The overall visual quality of samples was evaluated for the appearance of the entire sample. The aroma of samples was determined by opening the packages at the first time. For scoring, a 9-point rating scale was used to differentiate the changes of sample quality, where 1 = poor, 3 = fair, 5 = good and limit of marketability, 7 = very good, and 9 = excellent [3,4].

### 3.9. Statistical Analysis

The results were represented as means ± standard deviations and analyzed by analysis of variance (ANOVA) using SPSS (SPSS Inc., version 19.0, Chicago, IL, USA). Duncan’s multiple-range test was used to determine significant differences at 95% confidence level.

## 4. Conclusions

In conclusion, the incorporation of Ti and Ag nanoparticles into PLA packaging had antimicrobial effects on *T. aurantialba*. Compared to PLA, LDPE, PLA/REO films, *T. aurantialba* packed in PLA/REO/Ti and PLA/REO/Ti + Ag films showed better maintenance of tissue firmness and the overall acceptability, inhibition of microbial growth and vitamin C reduction, and preservation of the color of samples, although the effect on weight loss was not great. The results also suggested that PLA/REO/Ti and PLA/REO/Ti + Ag composite films were preferred for the application in *T. aurantialba* preservation and can extend the shelf life of *T. aurantialba* up to 16 days when stored at 4 ± 1 °C. However, the migration of nano-TiO_2_ and nano-Ag from the packaging films to *T. aurantialba* must be studied before the commercial use of the antimicrobial packaging could be recommended.
